# Trehalose enhances macrophage autophagy to promote myelin debris clearance after spinal cord injury

**DOI:** 10.1186/s13578-025-01357-2

**Published:** 2025-01-29

**Authors:** Zhida Ma, Congpeng Meng, Xiang Wang, Yuanzhe Zhao, Jingwen Wang, Yihao Chen, Yiteng Li, Yan Jiang, Fangru Ouyang, Jianjian Li, Meige Zheng, Li Cheng, Juehua Jing

**Affiliations:** 1 Department of Orthopaedics, The Second Affiliated Hospital of Anhui Medical University, Hefei, 230601 China; 2 Institute of Orthopaedics, Research Center for Translational Medicine, The Second Affiliated Hospital of Anhui Medical University, Hefei, 230601 China; 3 Department of Rehabilitation Medicine, The Second Affiliated Hospital of Anhui Medical University, Hefei, 230601 China

**Keywords:** Trehalose, Spinal cord injury, Foamy macrophages, Autophagy, Myelin debris

## Abstract

**Background:**

Myelin-laden foamy macrophages accumulate extensively in the lesion epicenter, exhibiting characteristics of autophagolysosomal dysfunction, which leads to prolonged inflammatory responses after spinal cord injury (SCI). Trehalose, known for its neuroprotective properties as an autophagy inducer, has yet to be fully explored for its potential to mitigate foamy macrophage formation and exert therapeutic effects in the context of SCI.

**Results:**

We observed that trehalose significantly enhances macrophage phagocytosis and clearance of myelin in a dose-dependent manner in vitro. In vivo, trehalose administration markedly reduced myelin debris accumulation, inhibited foamy macrophage formation, suppressed inflammatory responses, decreased fibrotic scarring, and promoted axonal growth and motor function recovery after SCI. These beneficial effects of trehalose may be related to the overexpression of transcription factor EB (TFEB), a key regulator of the autophagy-lysosomal system, which can rescue autophagic dysfunction in foamy macrophages and inhibit inflammatory responses. Additionally, the effects of trehalose on macrophages were abolished by chloroquine, an autophagy inhibitor, suggesting trehalose’s potential as a therapeutic candidate for enhancing myelin debris clearance post-SCI.

**Conclusions:**

Our findings underscore the pivotal role of trehalose in modulating myelin debris clearance within macrophages, providing new perspectives for the treatment of spinal cord injury.

**Graphical abstract:**

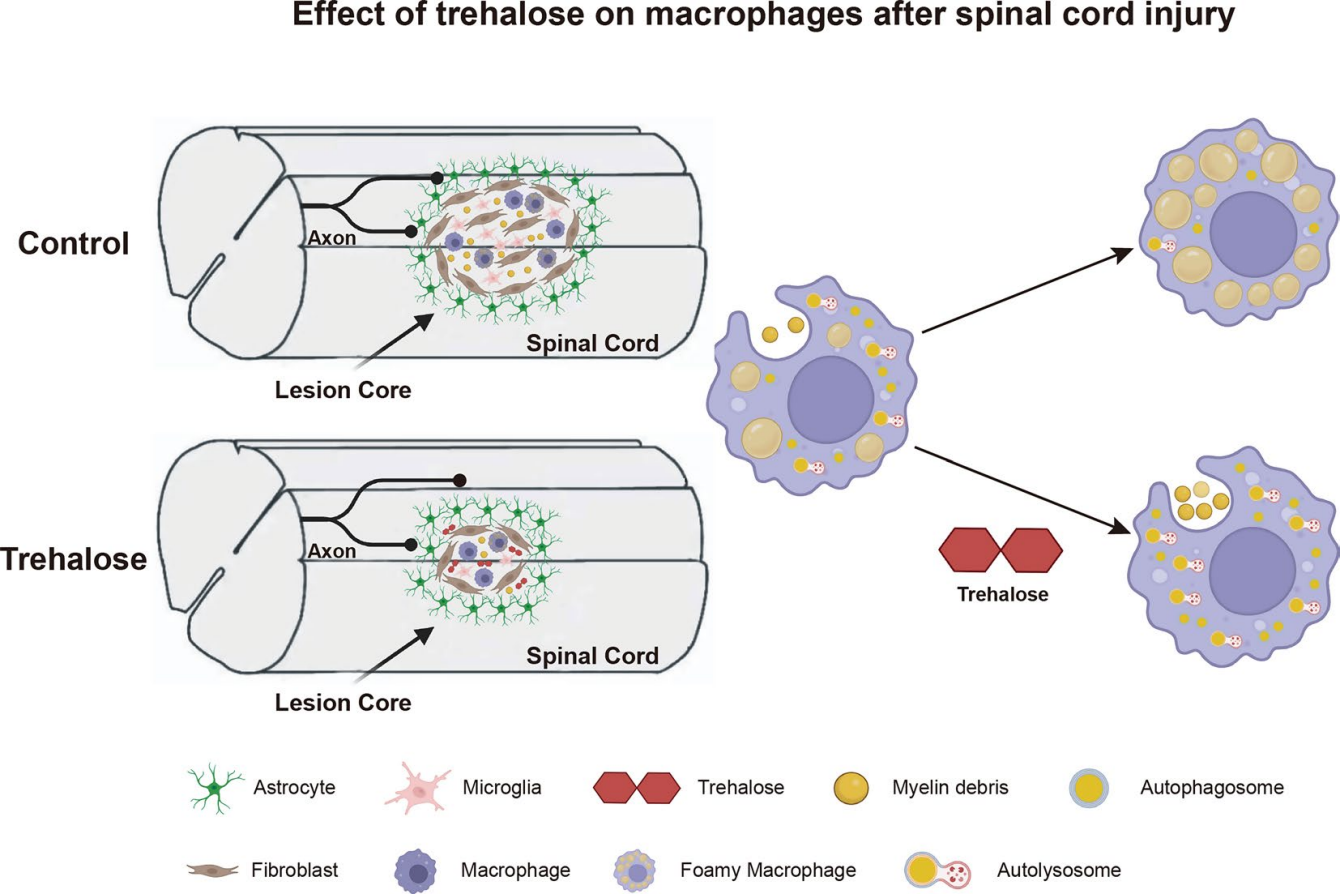

## Introduction

Spinal cord injury (SCI) represents a severe central nervous system (CNS) trauma characterized by extensive neuronal cell death and demyelination [1, 2]. The resultant myelin debris accumulation at lesion site impedes axonal remyelination and growth-cone formation [3–5]. Early infiltration of blood-derived macrophages into the lesion is crucial for phagocytosing this debris [6, 7]. However, extensive myelin ingestion results in the formation of foamy macrophages [8, 9], which exhibit diminished migratory and phagocytic capacities, reduced lipid efflux, and increased pro-inflammatory cytokine production [10, 11]. These foamy macrophages contribute to chronic inflammation, fibrotic scar formation, and impaired axonal regeneration, thereby hindering motor function recovery [12, 13]. Therefore, enhancing myelin debris clearance and reducing foamy macrophage formation are critical for improving recovery outcomes after SCI [14].

Autophagy, a vital cellular process for clearing intracellular waste and metabolizing substances such as accumulated lipids [15, 16], plays a significant role in cellular health. In atherosclerosis, disrupted macrophage autophagy impairs lipophagy and lysosome-mediated cholesterol efflux, leading to inflammasome hyperactivation and accelerated plaque formation. Conversely, enhancing autophagy can counteract these adverse effects [17–19]. Trehalose, a naturally occurring non-reducing disaccharide, is known to induce autophagy [20–22] and has shown promise in improving outcomes in neurodegenerative diseases by clearing aberrantly aggregated proteins in mouse models [23, 24]. Transcription factor EB (TFEB), a key factor of the autophagy-lysosomal system, regulates this process by increasing the expression of autophagy and lysosome-related genes [25–27]. In models of atherosclerosis and motor neuron degeneration, trehalose has been shown to boost autophagy by inducing TFEB overexpression [28, 29]. However, the effects of trehalose on macrophage TFEB expression, the autophagy-lysosome system, and SCI recovery remain unclear.

In this study, we demonstrated that trehalose enhances myelin debris clearance and reduces foamy macrophage formation after SCI by inducing TFEB expression in macrophages and modulating the autophagy-lysosomal system. By mitigating secondary inflammatory responses, trehalose decreased fibrotic scar and injury area sizes, thereby promoting axonal preservation and motor function recovery. These findings suggest that trehalose may represent a novel therapeutic approach for SCI.

## Materials and methods

### Animals and cells

All procedures involving animals were approved by the Ethics Committee of Anhui Medical University (Approval No. LLSC20211113) on October 12, 2021. Eight-week-old C57BL/6 female mice (weight 20–25 g) were purchased from the Animal Experimental Center of Anhui Medical University and randomly assigned to standard cages. The environment was temperature and humidity controlled, with food and water ad libitum on a 12-hour light/12-hour dark cycle. All experiments in this study were conducted and reported in accordance with the Animal Research: Reporting of In Vivo Experiments (ARRIVE) guidelines.

Mouse mononuclear macrophage leukemia cells (RAW 264.7), provided by the Stem Cell Bank of the Chinese Academy of Sciences (Shanghai), were cultured in Dulbecco’s modified Eagle’s medium (Cat#SH30021, HyClone, Logan, UT, USA) containing 1% glutamine, 1% sodium pyruvate (Invitrogen, USA) and 10% fetal bovine serum (Cat#10270106, Gibco, Grand Island, NY, USA)). The cells were cultured at 5% CO_2_ and 37 °C.

### Establishment of mouse spinal cord injury model

Animals were fasted (food and water) for 24 h prior to surgery. Mice were anesthetized by intraperitoneal injection of pentobarbital sodium (P-010; Sigma, St. Louis, Missouri, USA) at a dose of 50 mg/kg. The mice were placed in a supine position, their back hair was shaved, and the skin was disinfected. The T10 segment of the spinal cord was exposed by excising the lamina. A calibrated Dumont No. 5 forceps (11252-20, Fine Science Tools, Heidelberg, Germany) was used to completely clamp the T10 spinal cord from both sides for 5 s, resulting in a moderate compression spinal cord injury [30, 31]. The incisions were disinfected and sutured layer by layer. Routine anti-infection treatment and auxiliary urination care were provided twice a day after SCI.

### Trehalose treatment

In vivo, trehalose (T0176, SIGMA), dissolved in phosphate-buffered saline (PBS, BL601A, Biosharp, China) at a dose of 3 g/kg/day, was injected intraperitoneally 4 h after surgery. This treatment was continued for 14 days, while the control group was injected with equal volume of PBS.

In vitro, trehalose was prepared at four different concentrations: 0 mM, 10 mM, 20 mM, and 30 mM, and added to macrophages or foamy macrophages containing 0.5 mg/ml myelin. After determining the optimal concentration, 20 mM was used for next experiments. The control group received an equal amount of PBS, the experimental group received 20 mM trehalose, and the inhibition group received an equal amount of chloroquine (C6628, SIGMA) at a concentration of 30 mg/ml.

### Tissue preparation

After the mice were anesthetized, the hearts were exposed by thoracotomy and perfused with 0.1 M PBS followed by 4% paraformaldehyde (PFA, G1101, Servicebio, China). The spinal cord tissue, 0.5 mm in the lesion epicenter, was removed, immersed in 4% PFA for 4 h, and then placed in a 30% sucrose solution at 4 °C for dehydration until the tissue settled to the bottom. Subsequently, the tissue was cut into 16 μm-thick serial sagittal sections at -20 °C using a cryostat (NX50, Thermo Fisher Scientific, United States).

### Immunofluorescence staining

In vivo, sagittal slices containing the lesion area were used. Sections were dried at 50 °C for 1 h, washed three times with PBS, blocked for 1 h at room temperature with blocking solution, prepared in PBS containing 5% donkey serum (SL050, Solarbio, China) and 0.3% Triton X-100 (SL050 and T8200, Solarbio, China). Next, incubated overnight at 4 °C with the following primary antibodies: goat anti-5-hydroxy-tryptamine (5-HT) (1:5000, 20,079, Immunostar, United States), rat anti-F4/80 (1:100, 144801-82, Invitrogen, United States), rab anti-F4/80 (1:100, 28463-1-AP, Proteintech, China), rat anti-GFAP (1:400, 13–0300, Thermo Fisher Scientific, United States), rabbit anti-degraded myelin basic protein (dMBP) (1:100, AB5864, Chemicon, United States), rabbit anti-NeuN (1:500, ab177487, Abcam, United Kingdom), rat anti-Lamp1(1:100, 14-1071-82, Invitrogen, United States), rab anti-Lamp1 (1:100, 9091S, Cell Signaling Technology, United States), rat anti-CD68 (1:400, MCA1957, Bio-Rad, United States), rabbit anti-Fibronectin (1:100, 15613-1-AP, Proteintech, China), rabbit anti-Laminin (1:100, 23498-1-AP, Proteintech, China), rabbit anti-neurofilament heavy polypeptide (NFH) (1:500, ab207176, Abcam, United Kingdom), goat anti-PDGFRβ (5 µg/ml, AF1042-SP, R&D Systems, United States), rabbit anti-GFAP (1:100, 16825-1-AP, Proteintech, China), rabbit anti-LC3B (1:200, Abcam, ab192890, United Kingdom), mouse anti-P62 (1:500, 66184-1-Ig, Proteintech, China), rabbit anti-TFEB (1:100, 13372-1-AP, Proteintech, China). The sections were then washed 4 times with PBS and incubated with the following secondary antibodies for 1 h at room temperature in the dark: donkey anti-rabbit Alexa Fluor 555 (1:500, A-31572, Thermo Fisher Scientific, United States), donkey anti-rabbit Alexa Fluor 488(1:500, A-21206, Thermo Fisher Scientific, United States), donkey anti-rat Alexa Fluor 488(1:500, A-21208, Thermo Fisher Scientific, United States), donkey anti-goat Alexa Fluor 488 (1:500, A-11055, Thermo Fisher Scientific, United States), donkey anti-mouse Alexa Fluor 488(1:500, A-31570, Thermo Fisher Scientific, United States) donkey anti-goat Alexa Fluor 555 (1:500, A-21432, Thermo Fisher Scientific, United States) and donkey anti-rat Alexa Fluor 555 (1:500, A48270, Thermo Fisher Scientific, United States). Finally, nuclei were stained with 4ʹ6-diamidino-2-phenylindole (DAPI, C1005, Beyotime Biotech, China). Negative control sections were incubated with secondary antibody only.

In vitro, the cell culture slides were fixed with 4% PFA for 15 min, washed three times with PBS, and then blocked with 5% donkey serum albumin (DSA, Solarbio, SL050) for 30 min. The primary antibody was incubated overnight at 4 °C. The slides were then washed three times with PBS and incubated with secondary antibody for 1 h at room temperature in the dark. Finally, the slides were sealed with DAPI, and representative images were obtained by comparative analysis using fluorescence microscopy.

### Preparation of myelin debris and dio-myelin

Myelin debris was extracted from 8 to 12-week-old mice. After euthanasia, the brain tissue was removed and homogenized with ice-cold 0.32 M sucrose. The myelin debris solution was purified by sucrose density gradient centrifugation using ultra-high-speed centrifuges [14]. The prepared myelin debris solution was then labeled with 3,3’-Dioctadecyloxacarbocyanine perchlorates (Dio, C1993S, Beyotime Biotechnology, China) and incubated it in the dark at 37 °C for 20 min. The final Dio-myelin preparation was successful [32].

### Oil red O (ORO) staining

ORO staining was performed using Oil Red O staining kit (G1262, Solarbio, China). ORO staining detects myelin debris accumulated in the lesion area and in RAW 264.7 cells. In the tissue experiment: the spinal cord sections were dried, stained with ORO for 10 min, soaked in 60% isopropanol for 3 min, and washed with distilled water. Representative images were obtained under a fluorescence microscope (AxioScopeA1, Zeiss, Germany). In cell experiment: cell coverslips were washed with PBS, fixed with ORO fixative solution for 15 min, and dehydrated with 60% isopropanol for 4 min. The 60% isopropanol were removed, and freshly prepared ORO staining solution was added for 10 min. The stain was removed, and the coverslips were washed 6 times with PBS until there is no excess. Mayer’s hematoxylin staining solution was then added for 1 min. The dye was removed, and the coverslips were washed 6 times with PBS. Cell coverslips were soaked in ORO buffer for 1 min and then observed under a microscope [33, 34].

### Image acquisition and quantitative analysis

Representative images were obtained by comparison and analysis using a fluorescence microscope (AxioScopeA1, Zeiss, Germany), ensuring consistent light intensity across all images. All quantitative analyses were performed in a blinded fashion. Quantitative image analysis was conducted with Image J version 2.0 (National Institutes of Health, Bethesda, MD, USA). To assess the accumulation of myelin debris in foamy macrophages, these cells were classified into three levels based on the proportion of lipid droplets occupying the cytoplasm as < 1/3, 1/3 − 2/3 and > 2/3, respectively. The percentage of cells with lipid droplet content greater than 1/3 was quantified [35]. The number of cells in each group (*n* ≥ 60 cells per group) was counted using a Zeiss microscope and ZEN imaging software. Statistical analysis was performed using the sum of the proportions of lipid droplets occupying the cytoplasm and the cells [33–35]. To quantify myelin debris accumulation in the lesion area after SCI, the proportion of dMBP^+^ area relative to the GFAP^−^ area was calculated. The ratio of ORO^+^ area to the observed field of view was used to quantify foamy macrophage formation.

Tissue recovery was assessed by calculating the ratio of the quantified GFAP^−^ area to the observed field of view. The inflammatory areas were evaluated by quantifying the immunoreactive positive areas of CD68, and the areas of fibrotic scarring were evaluated by quantifying the immunoreactive positive areas of PDGFRβ, Laminin, and Fibronectin. These measurements were normalized to 4× images of sections spanning the lesion area [36]. Axonal preservation was evaluated by selecting a 300 μm long boxed area centered on the lesion epicenter, quantifying the area of NFH^+^ neurofilaments within this region, and calculating the ratio to the total area. Neuronal survival was assessed by delineating three regions (Z1-Z3), each 250 μm long, at lesion epicenter of spinal cord, and quantifying the density of NeuN^+^ neurons in these regions. To evaluate axonal preservation, a quantitative analysis of the area of the 5-HT^+^ axons in the epicenter and the rostral side was conducted. At least 4 animals were examined in each group of the above experiments, and the section across the lesion area and two adjacent sections 180 μm apart were selected for staining, and the results were quantified by averaging [1].

In 40× images across the lesion area, dMBP^+^ macrophages at 7, 14 and 28 days were counted to evaluate their myelin phagocytosis ability. LC3B^+^ and P62^+^ macrophages were counted to assess macrophage autophagy, and Lamp1^+^ macrophages were counted to evaluate lysosomal function. The final results for each sample were averaged using 3 random 40× images, with a least 4 animals per group.

### Behavioral assessments

All behavioral assessments were conducted in a blinded manner to ensure unbiased results.

The Basso Mouse Scale (BMS) is commonly used to evaluate the recovery of motor function in mice after SCI. Scores range from 0 (no ankle movement) to 9 (full functional recovery), and movement was scored based on hindlimb joint movement, toe clearance, trunk position and stability, paw position, pace coordination, and tail position. The BMS in this study was conducted in an open field according to the protocol developed by Basso and colleagues. All mice received BMS evaluations before SCI to confirm normal motor function and after surgery to validate successful SCI modeling. All mice were observed and evaluated by three independent researchers at 0, 3, 7, 14, 21, and 28 days post-injury (dpi) with 5 animals per group, and the final results were averaged [37].

Footprint analysis was used to further evaluate gait recovery and motor coordination in mice at 28 dpi. Different colored dyes were applied to the front and hind paws. Step length was measured as the distance from the starting point to the endpoint of the hind paw in a gait cycle. Stride width was determined by the distance from the outermost toe of the left paw to the outermost toe of the right paw. Paw rotation was assessed by the angle between the body centerline axis and the rear paw axis. All evaluations included more than three consecutive gait cycles on each side, with an average of 5 animals per group. Mice without SCI were included in the uninjured group for comparison [38, 39]. Footprints were digitized, and representative images were used to evaluate coordination.

### Statistical analysis

Each group of experiments was independently repeated in at least 4 animals, 3 sections in each sample were stained, and the data were expressed as the mean ± standard error of the mean (SEM). Analysis one-way or two-way analysis of variance (ANOVA), followed by a post hoc Tukey-Kramer test, was used to compare differences among multiple groups. Comparisons between two groups were performed using Student’s *t* test. Data analysis and graphing were conducted using GraphPad Prism 8.3.2 software (GraphPad, United States). Significance was reported as ns, no significance, ^*^p < 0.05, ^**^p < 0.01, ^***^p < 0.001 or ^****^p < 0.0001.

## Results

### Trehalose enhances macrophage phagocytosis of myelin debris in vitro with an optimal concentration of 20 mM

**Fig. 1:**
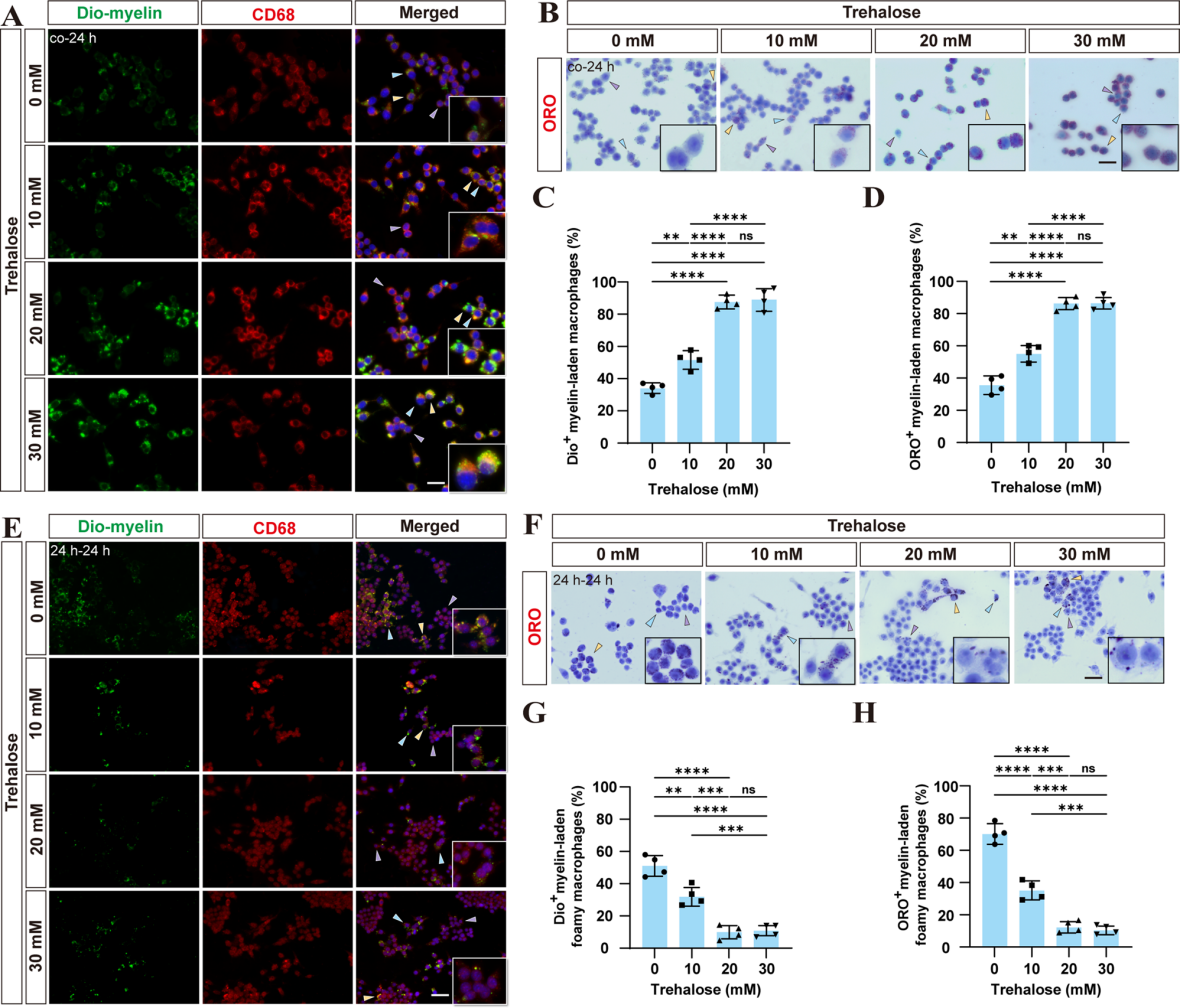
Trehalose promotes both the phagocytosis of myelin debris by macrophages and the expulsion of myelin debris from foamy macrophages in vitro. **A** Macrophages (CD68^+^, red) were labeled using immunofluorescence staining. **B** Myelin debris was labeled with ORO staining (red), and nuclei with hematoxylin. Purple, blue and yellow arrows indicate typical macrophages with myelin debris occupying less than 1/3, between 1/3 and 2/3, and more than 2/3 of the cytoplasm, respectively. Scale bars for both **A** and **B**, 40 μm. **C**, **D** Quantitative analysis compared the proportion of macrophages with more than 1/3 of their cytoplasm occupied by myelin debris across different trehalose treatment groups. Results are presented as mean ± SEM. ^**^p < 0.01, ^****^p < 0.0001 and ns, no significance by one-way ANOVA. **E** Macrophages (CD68^+^, red) were labeled using immunofluorescence staining. Scale bars, 100 μm. **F** Myelin debris was labeled with ORO staining(red) and nuclei with hematoxylin. Scale bars, 40 μm. **G**, **H** Quantitative analysis of the percentage of foamy macrophages with more than 1/3 of their cytoplasm occupied by myelin debris across different trehalose treatment groups. Results are presented as mean ± SEM. ^**^p < 0.01, ^***^p < 0.001, ^****^p < 0.0001 and ns, no significance by one-way ANOVA. Co-24 h in **A** and **B** means RAW 264.7 cells were incubated with 0.5 mg/ml Dio-myelin debris and varying trehalose concentrations for 24 h. 24 h–24 h in **E** and **F** means cells were incubated with medium containing 0.5 mg/ml Dio-myelin debris (green) for 24 h and then treated with different concentrations of trehalose for 24 h

We first examined the effect of trehalose on macrophage phagocytosis of myelin debris *in vitro.* It is known that macrophage phagocytic capacity saturates at 0.5 mg/ml myelin debris [35]. To investigate whether trehalose can further affect the phagocytosis of myelin debris in macrophages that have already undergone phagocytic saturation, macrophages were incubated with varying concentrations of trehalose and 0.5 mg/ml of Dio-dye-labeled myelin debris. Immunofluorescence staining results indicated that the proportion of myelin debris within macrophages increased with rising trehalose concentrations, reaching a peak at 20 mM. Beyond this concentration, the phagocytic capacity did not significantly change (Fig. 1A, C). ORO staining confirmed these findings, showing that the proportion of neutral lipids, which are myelin degradation products, in macrophages increased up to 20 mM trehalose, with no further change observed (Fig. 1B, D). Thus, trehalose enhances macrophage phagocytosis of myelin debris, optimally at 20 mM.

### Trehalose promotes the processing of myelin debris in foamy macrophages in vitro at an optimal concentration of 20 mM

Next, we investigated the effect of trehalose on foamy macrophages in vitro. Initially, macrophages were cultured with 0.5 mg/ml of Dio-dye-labeled myelin debris for 24 h to establish a foamy macrophage model [35]. Subsequently, the impact of varying concentrations of trehalose on these foamy macrophages was assessed. Immunofluorescence analysis showed that myelin debris within foamy macrophages gradually decreased with higher trehalose levels, stabilizing at 20 mM (Fig. 1E, G). ORO staining confirmed these results. At 20 mM trehalose, macrophages effectively manage accumulated myelin debris in vitro (Fig. 1F, H).

### Trehalose treatment enhances lipid phagocytosis and facilitates lipid processing by foamy macrophages after SCI

**Fig. 2:**
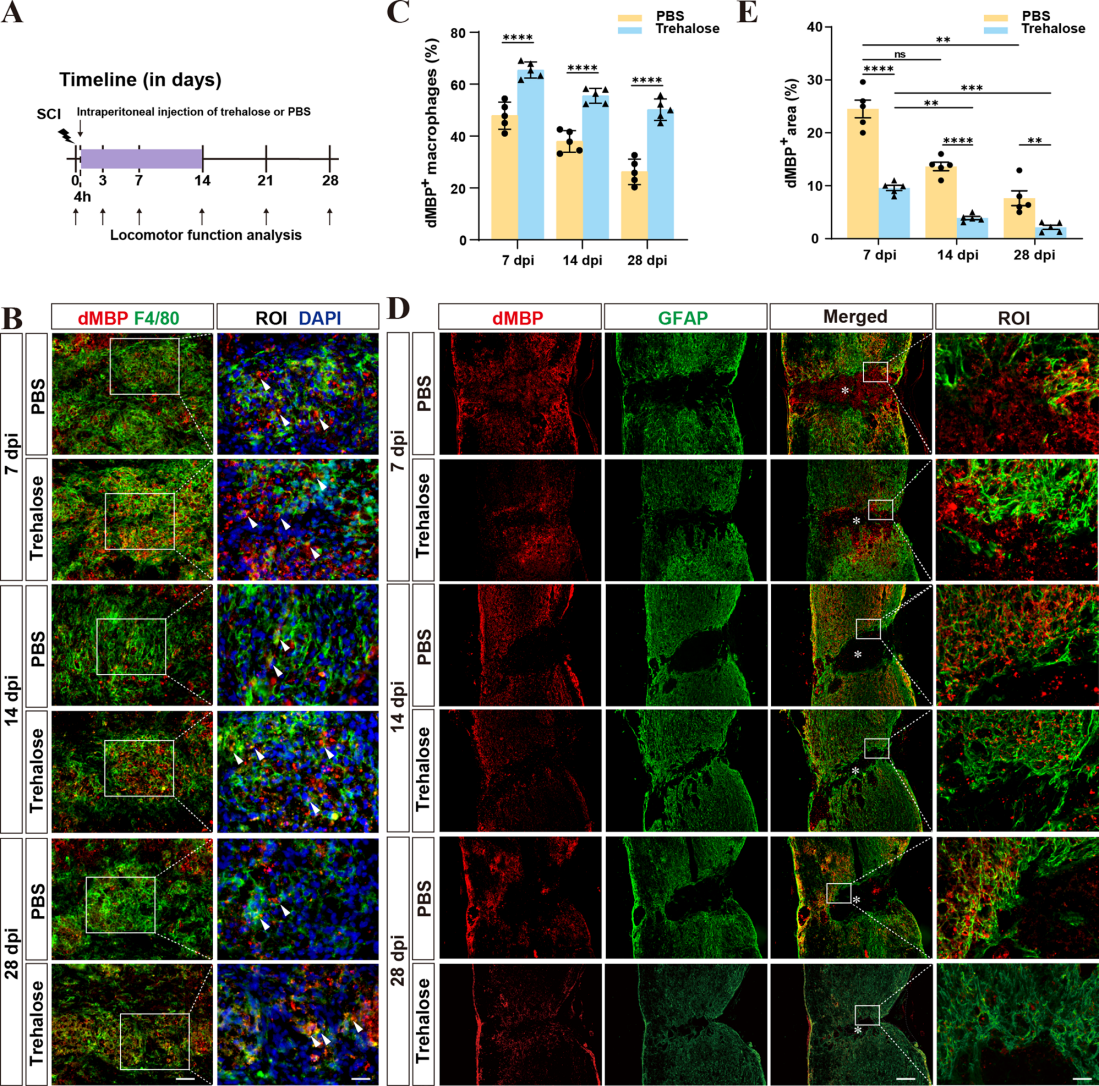
Trehalose promotes lipid phagocytosis and clearance of myelin debris from the lesion area after SCI. **A** Timeline of the intraperitoneal injection and the behavioral assessment. **B** Representative images of dMBP (red) and F4/80 (green) immunofluorescence staining in the PBS and Trehalose treatment groups at 7, 14, and 28 dpi. Scale bars: 40 μm (low magnification images), 20 μm (high magnification images). **C** Quantification of the proportion of dMBP^+^ macrophages in the PBS and Trehalose treatment groups at 7, 14, and 28 dpi. ^****^p < 0.0001 by two-way ANOVA. **D** Representative images of dMBP (red) and GFAP (green) immunofluorescence staining at 7, 14, and 28 dpi. Scale bars: 200 μm (low magnification images), 20 μm (high magnification images). **E** Quantitative analysis of the dMBP^+^ area in the lesion area of the PBS and Trehalose treatment groups at each time point after SCI. ns, no significance. ^**^p < 0.01, ^***^p < 0.001, and ^****^p < 0.0001 by two-way ANOVA. *n* = 5 animals in **C** and **E**. The asterisks indicate the lesion epicenter. The region of interest (ROI) represents the boxed region on the left

**Fig. 3:**
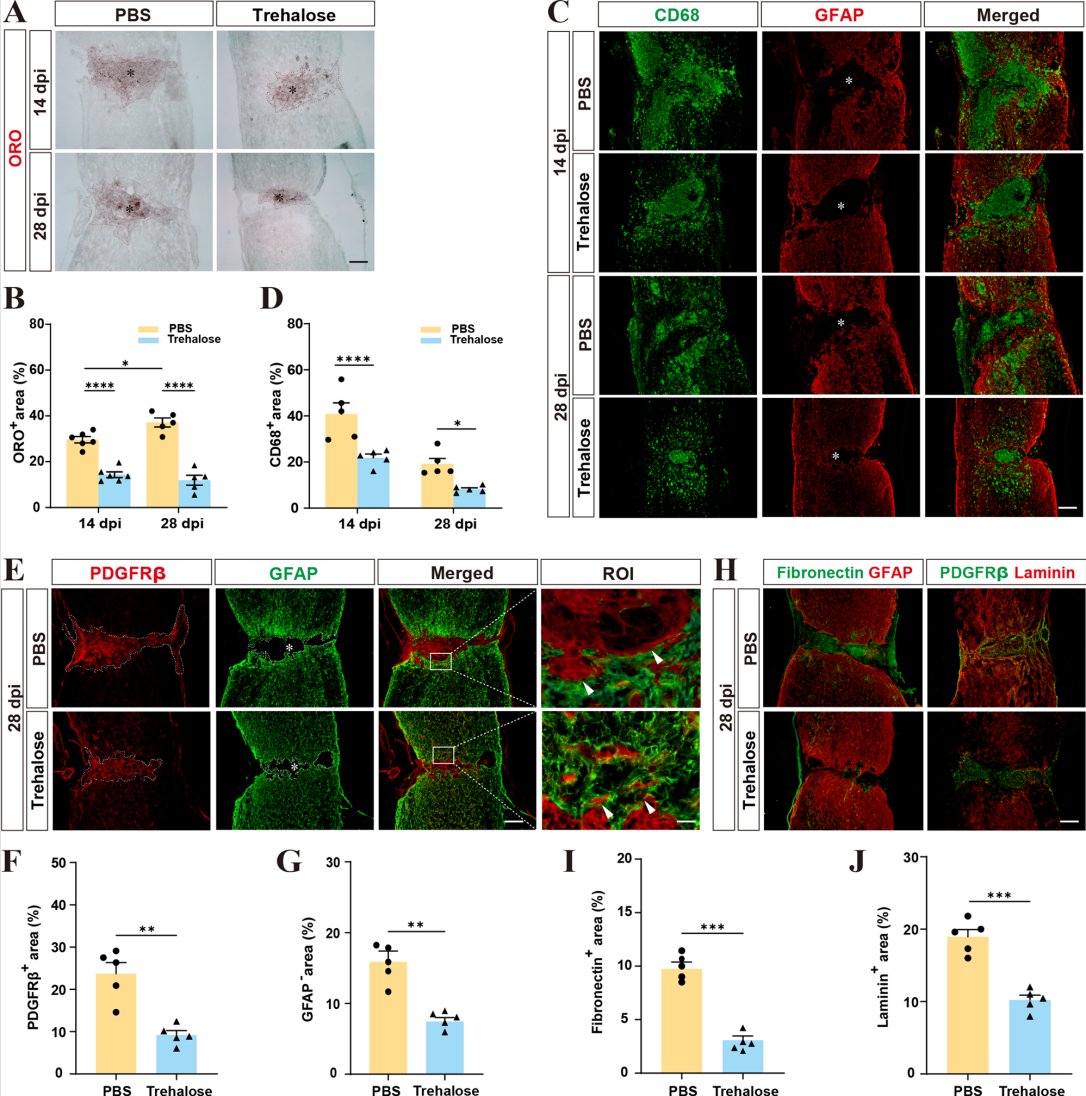
Trehalose can inhibit the inflammatory response after SCI, reduce the area of injury, and inhibit the formation of fibrotic scars. **A** Representative images of ORO staining of lesion areas in the PBS and Trehalose treatment groups at 14 and 28 dpi. **B** Quantitative analysis of the ORO^+^ area in the PBS and Trehalose treatment groups at 14 and 28 dpi. ^*^p < 0.05 and ^****^p < 0.0001 by two-way ANOVA. **C** Representative images of GFAP (red) and CD68 (green) at 14 and 28 dpi. **D** Quantitative analysis of the CD68^+^ area in the PBS and Trehalose treatment groups at 14 and 28 dpi. ^*^p < 0.05 and ^****^p < 0.0001 by two-way ANOVA. **E** Immunofluorescence staining of PDGFRβ (red) and GFAP (green) in sagittal sections of the PBS and Trehalose treatment groups at 28 dpi. ROI represents the boxed region on the left and shows the fibrotic/astrocytic scar boundary. **F**, **G** Quantitative analysis of the PDGFRβ^+^ and GFAP^−^ area in the PBS and Trehalose treatment groups at 28 dpi. ^**^p < 0.01 by Student’s *t* test. **H** Representative images of Fibronectin (green) and GFAP (red) at 28 dpi. Representative images of Laminin (red) and PDGFRβ (green) at 28 dpi. **I**, **J** Quantitative analysis of the Fibronectin^+^ area and Laminin^+^ area in the PBS and Trehalose treatment groups at 28 dpi. ^***^p < 0.001 by Student’s *t* test. *n* = 5–6 animals in **B**, **D**, **F**, **G**, **I**, and **J**. The asterisks indicate the lesion epicenter. Scale bars: 200 μm (low magnification images), 20 μm (high magnification images)

Demyelination changes occur after SCI, forming large amounts of myelin debris and triggering secondary inflammatory responses. Macrophages, as primary phagocytes, key in the phagocytosis of myelin debris [32, 40]. The transformation of myelin debris-consuming macrophages into foamy macrophages can lead to tissue necrosis and chronic inflammation. Reducing the formation of foamy macrophages aids SCI recovery [41, 42]. We thus investigated the trehalose’s effect on myelin debris clearance by macrophages after SCI. Trehalose (3 g/ kg/ day) [20] was injected intraperitoneally starting 4 h after SCI until day 14. The groups were analyzed for rehabilitation status post-injury (Fig. 2A). Myelin basic protein was specifically labeled with dMBP and the GFAP^−^ region was used to represent the lesion epicenter [43]. Macrophages were labeled with F4/80 [44]. At 7, 14, and 28 dpi, staining revealed that F4/80^+^ macrophages in the trehalose treatment group exhibited greater colocalization with dMBP^+^ myelin basic protein compared to the control group (Fig. 2B, C). Fluorescence results showed that the levels of dMBP^+^ myelin basic protein in the lesion area of control group mice were significantly higher than those in the trehalose treatment group (Fig. 2D, E). This suggests trehalose enhances debris clearance or reduces foamy macrophage formation. Thus, we explored the effect of trehalose treatment on the formation of foamy macrophages. The accumulation of foamy macrophages in lesion areas was observed using ORO staining [33, 34], where positive staining for ORO is indicative of foamy macrophages [42]. The results showed that the ORO^+^ area in the trehalose treatment group at 14 and 28 dpi was significantly smaller than that in the control group (Fig. 3A, B). This indicates that foamy macrophages are abundant and persist long-term after SCI, and that trehalose treatment can reduce the formation of foamy macrophages in the spinal cord lesions.

### Trehalose treatment reduces inflammation and fibrotic scar formation after SCI

Due to the presence of foamy macrophages, which lead to tissue necrosis and chronic inflammation [42], we focused on the inflammatory responses during the chronic phase of SCI. Our results indicated that at 14 and 28 dpi, the area of CD68^+^ inflammatory cells in the trehalose-treated group was significantly reduced compared to the control group (Fig. 3C, D). This reduction suggests that decreasing foamy macrophage formation alleviates chronic inflammation post-SCI, and this is consistent with the results of previous studies [36]. Following SCI, perivascular fibroblasts proliferate, migrate, and deposit a large amount of extracellular matrix (ECM), including fibronectin and laminin. They eventually form fibrous scars that encapsulate macrophages at the lesion epicenter and closely associate with astrocytes at the injury margin, hindering axonal regeneration and functional recovery [8, 45]. The inflammatory responses promote the formation of fibrotic scars post-SCI, exacerbating the injury [36]. Therefore, we used immunofluorescence to detect PDGFRβ, fibronectin, and laminin to assess changes in fibroblasts and fibrotic ECM, detected GFAP to evaluate changes in astrocytic scars [46], and used GFAP-negative areas to represent the lesion size [1]. At 28 dpi, the trehalose-treated group had interwoven fibrotic and astrocytic scars with indistinct boundaries, unlike the control group, which had dense, continuous fibrotic scars (Fig. 3E-G). Additionally, the area of fibrotic scars, including PDGFRβ^+^ fibroblasts and fibrotic EMC indicated by the positive of fibronectin or laminin, was significantly reduced in the trehalose-treated group compared to the control group (Fig. 3H-J). The widely accepted view is that the dense, continuous boundaries formed by the excessive deposition of fibroblasts post-SCI are one of the main reasons hindering axonal regeneration and functional recovery [44, 46]. Therefore, our study suggests that trehalose treatment can alleviate chronic inflammation post-SCI, effectively inhibit fibrotic scar formation, and promote the formation of astrocytic scars, thus providing a favorable internal environment for SCI recovery.

### Trehalose treatment enhances neuron survival, axonal preservation and motor function recovery after SCI

**Fig. 4:**
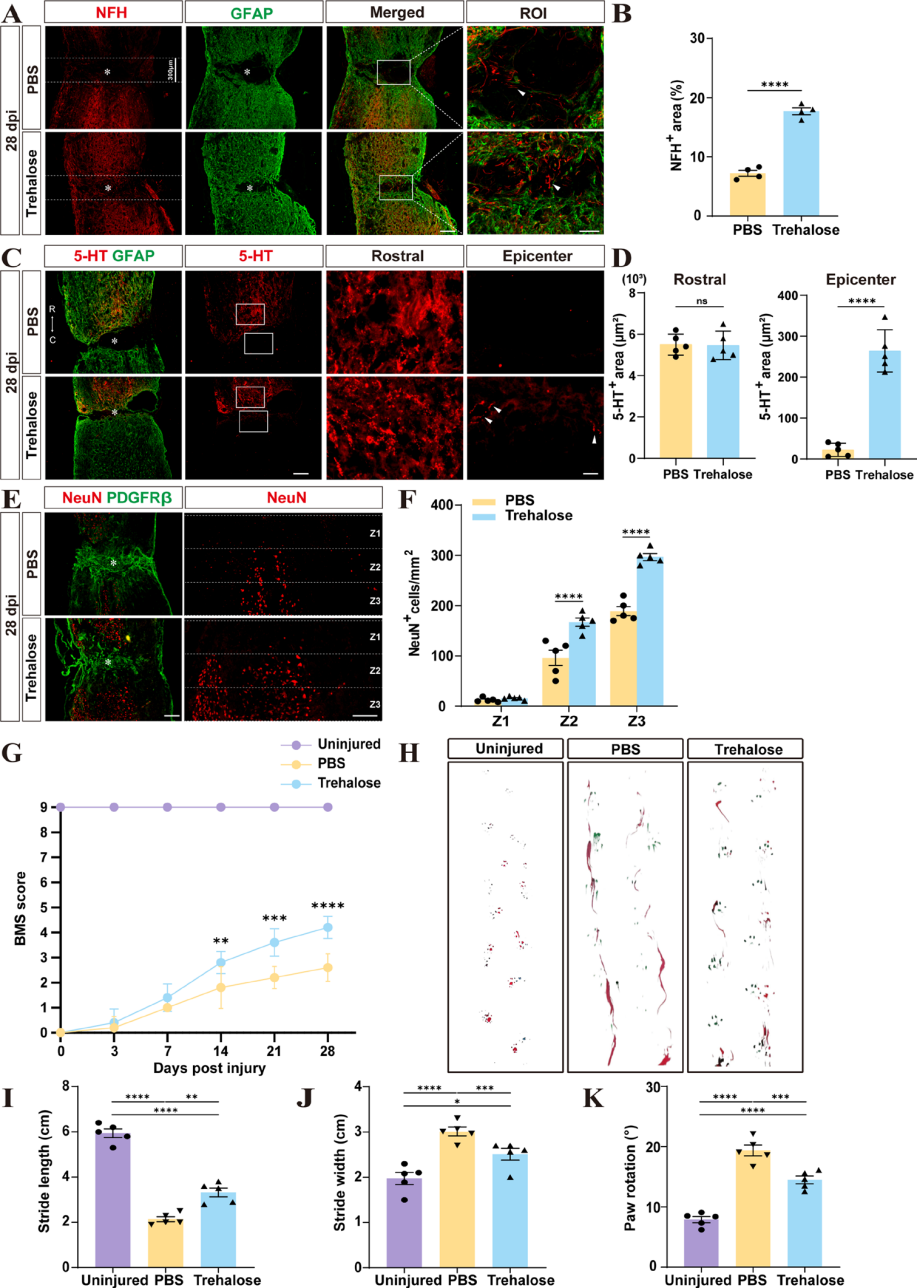
Trehalose promotes axonal preservation and motor function recovery after SCI in mice. **A** Representative images of NFH (red) and GFAP (green) immunofluorescence staining at 28 dpi. ROI represents the boxed area on the left, showing detailed immunostaining of NFH^+^ neurofilaments and GFAP. The arrows point to typical NFH^+^ neurofilaments in the lesion epicenter. **B** Quantitative analysis of the proportion of NFH^+^ neurofilament area to dotted line sealing area showed that 150 μm areas on both sides of the lesion epicenter were separated by dotted lines. ^****^p < 0.0001 by Student’s *t* test. **C** Representative images of 5-HT (red) and GFAP (green) immunofluorescence staining at 28 dpi. Rostral and epicenter represents the square area on the left, showing detailed immunostaining of 5-HT. The arrows point to typical 5-HT^+^ axons. R and C represents the rostral side and the caudal side of spinal cord. **D** Quantitative analysis of 5-HT^+^ axons area in rostral or epicenter. ns, no significance. ^****^p < 0.0001 by Student’s *t* test. **E** Representative images of NeuN (red) and PDGFRβ (green) immunofluorescence staining at 28 dpi. **F** Quantification of the mean density of NeuN^+^ cells in the dotted line sealed area (Z1-Z3) in two groups. ^****^p < 0.0001 by Student’s *t* test. **G** Locomotor function was evaluated by BMS at 0, 3, 7, 14, 21, and 28 dpi. ^**^p < 0.01, ^***^p < 0.001, and ^****^p < 0.0001 (PBS vs. Trehalose group at 14, 21, and 28 dpi) by two-way ANOVA. **H** Representative images of footprint analysis in the Uninjured, PBS and Trehalose treatment groups at 28 dpi. The hind paws are shown in red dye, and the front paws are shown in green dye. **I-K** Quantification of the stride length, stride width and paw rotation at 28 dpi. ^*^p < 0.05, ^**^p < 0.01, ^***^p < 0.001, and ^****^p < 0.0001 by one-way ANOVA. *n* = 4–5 animals in **B**, **D**, **F**, **G**, **I**, **J** and **K**. Asterisks indicate the epicenter of the lesion. Scale bar: 200 μm (low power image), 40 μm (high power image)

To validate trehalose’s effects on axonal preservation following SCI, we employed immunofluorescence staining to assess NFH fibers and the descending serotonergic 5-HT axon. The results indicated a significant increase in NFH fibers density in the trehalose-treated group (Fig. 4A, B). Additionally, the trehalose-treated group showed a marked increase in the area of 5-HT axons, with 5-HT axons extending across the lesion area from the rostral to the caudal side, in contrast to the control group where 5-HT axons failed to cross the lesion area (Fig. 4C, D) [47]. Immunofluorescence staining revealed that the NeuN^+^ neuron density in the lesion epicenter and surrounding areas (Z1-Z3) was significantly higher in the trehalose-treated group compared to the control group (Fig. 4E, F) [48], suggesting that trehalose enhances the survival of NeuN^+^ neurons in the lesion epicenter and promotes the preservation of NFH fibers. These findings collectively imply that trehalose treatment facilitates axonal preservation and neuronal survival post-SCI, potentially due to a reduction in foamy macrophage formation and suppression of inflammation and fibrotic scar formation. Enhanced axonal preservation and neuronal survival are typically associated with better motor function recovery. Therefore, we further assessed the motor function recovery in mice using the BMS score and footprint analysis. The BMS scores of the uninjured group was always 9 points. No statistically significant differences in BMS scores were observed between the trehalose-treated group and the PBS group before 7 dpi. However, the trehalose-treated group exhibited significantly better hindlimb motor function recovery and higher BMS scores at 14, 21, and 28 dpi compared to the PBS group (Fig. 4G). Footprint analysis at 28 dpi demonstrated superior motor function recovery in the trehalose-treated group, including narrower paw rotation, longer stride length, and increased step frequency (Fig. 4H-K). These results indicate that trehalose significantly enhances neuron survival, axonal preservation, and motor function recovery after SCI.

### Trehalose treatment enhances autophagic lysosomal function and TFEB expression in macrophages after SCI

**Fig. 5:**
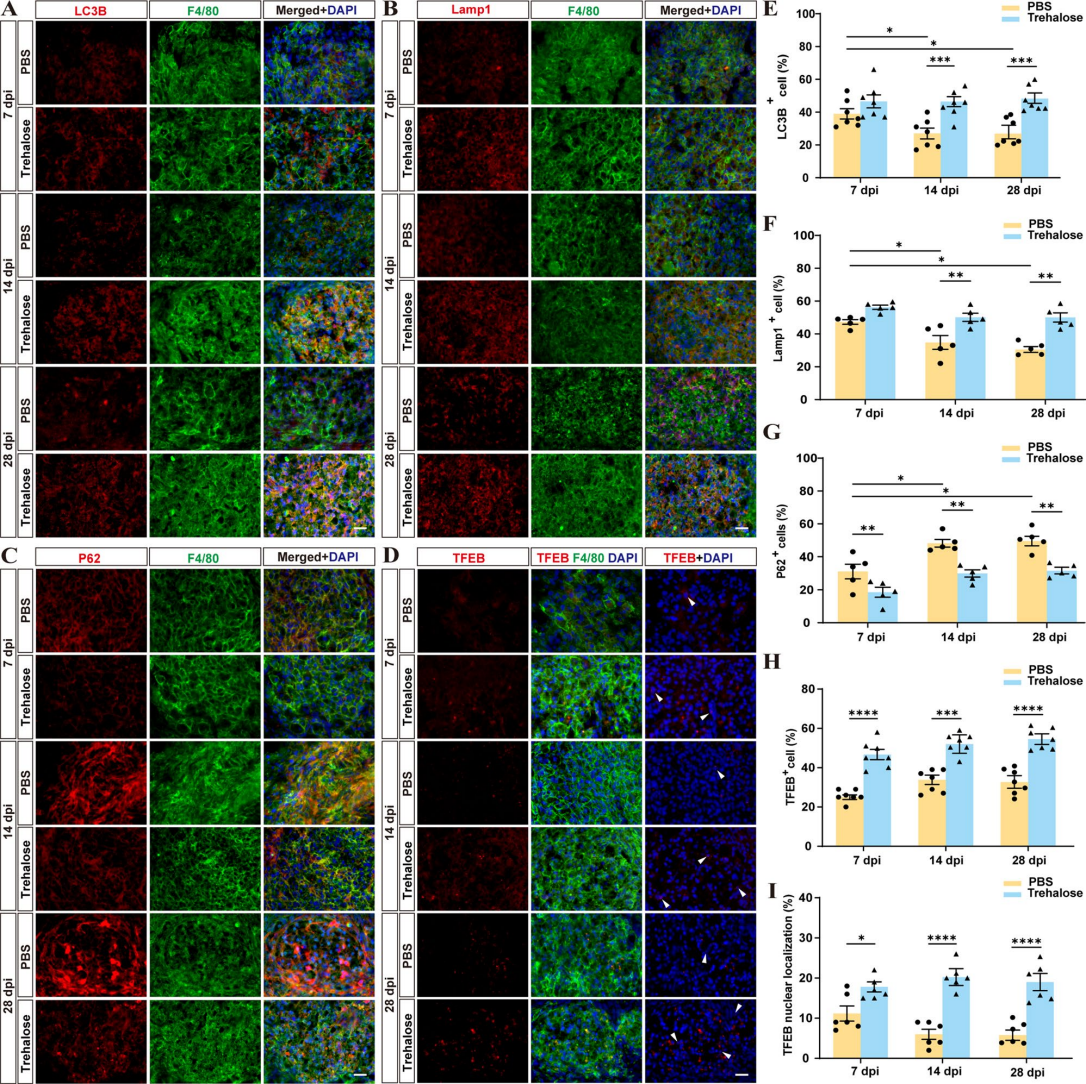
Trehalose enhances autophagic-lysosomal function and promotes the expression of TFEB in macrophages after SCI in mice. **A** Representative images of LC3B (red) and F4/80 (green) immunofluorescence staining within the lesion area at 7, 14 and 28 dpi. Mice were injected intraperitoneally with PBS and trehalose. **B** Representative images of Lamp1 (red) and F4/80 (green) immunofluorescence staining within the lesion area at 7, 14 and 28 dpi. **C** Representative images of P62 (red) and F4/80 (green) immunofluorescence staining within the lesion area at 7, 14 and 28 dpi. **D** Representative images of TFEB (red) and F4/80 (green) immunofluorescence staining within the lesion area at 7, 14 and 28 dpi. The white arrows indicate that TFEB is located in the nucleus. **E** Quantification of the proportion of LC3B^+^ macrophages in macrophages in **A**. ^*^p < 0.05 and ^***^p < 0.001 by two-way ANOVA. **F** Quantification of the proportion of Lamp1^+^ macrophages in macrophages. ^*^p < 0.05 and ^**^p < 0.01 by two-way ANOVA. **G** Quantification of the proportion of P62^+^ macrophages in macrophages. ^*^p < 0.05 and ^**^p < 0.01 by two-way ANOVA. **H** Quantification of the proportion of TFEB^+^ macrophages in macrophages. ^***^p < 0.001 and ^****^p < 0.0001 by two-way ANOVA. **I** Quantification of the proportion of TFEB nuclear localization. ^*^p < 0.05 and ^****^p < 0.0001 by two-way ANOVA. *n* = 5–7 animals in **E**-**I**. All results are shown as the means ± SEM. Scale bar: 20 μm

**Fig. 6:**
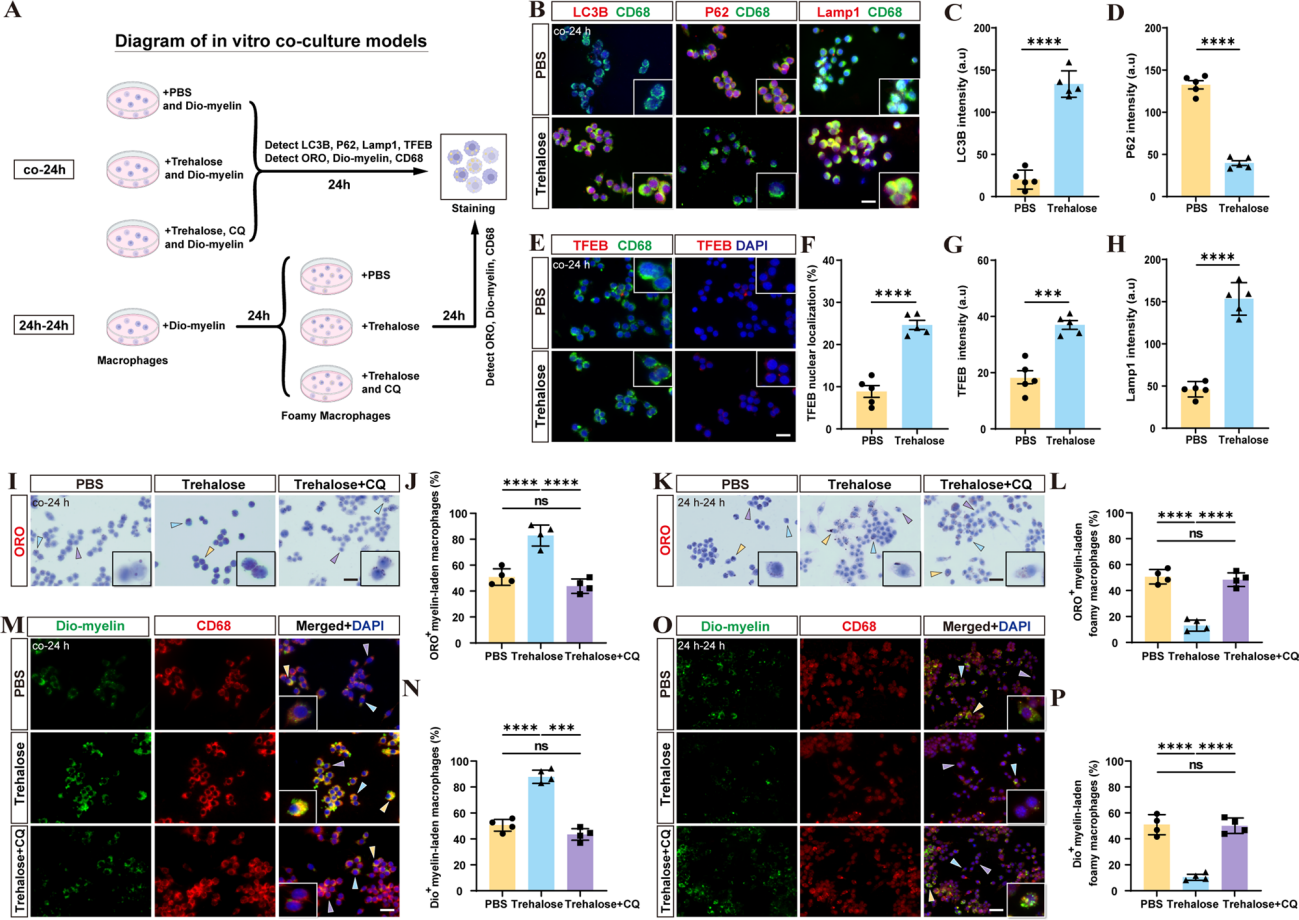
Trehalose enhances autophagic-lysosomal function, promotes the expression of TFEB and the clearance of myelin debris in macrophages in vitro. **A** Schematic diagram of the working model of in vitro co-culture. **B** RAW 264.7 cells were incubated with myelin and 20 mM trehalose for 24 h. The same volume of PBS was used for the control group. Intracellular levels of LC3B, P62 and Lamp1 (red) were detected by immunofluorescence, with RAW 264.7 cells labeled with CD68 (green). **C**, **D**, **H** Quantification of the mean LC3B, P62 and Lamp1 intensity per cell in **B**. ^****^p < 0.0001 by Student’s *t* test. *n* ≥ 60 cells in each group. **E** Detection of intracellular levels of TFEB (red) was performed by immunofluorescence, with RAW 264.7 cells labeled with CD68 (green). Nuclei were labeled with DAPI (blue). **F** Quantification of TFEB localization with nucleus for each cell in **E**. ^****^p < 0.0001 by Student’s *t* test. *n* ≥ 60 cells in each group. **G** Quantification of the mean TFEB intensity per cell in **E**. ^***^p < 0.001 by Student’s *t* test. *n* ≥ 60 cells in each group. **I**, **K**, **M**, **O** RAW 264.7 cells were treated with 0.5 mg/ml Dio-myelin debris, and 20 mM trehalose or chloroquine in a manner of co-24 h (I, M) or 24–24 h (K, O). Myelin debris were labeled with ORO stain (red) and nuclei were labeled with hematoxylin in **I** and **K**. Immunofluorescence staining labeled RAW 264.7 cells (CD68^+^, red) in **M** and **O**. Purple, blue and yellow arrows indicate typical macrophages with myelin debris occupying less than 1/3, between 1/3 and 2/3, and more than 2/3 of the cytoplasm, respectively. **J**, **L**, **N**, **P** Statistical analysis compared the proportion of cells with more than 1/3 of Dio-myelin or ORO^+^ myelin debris in the cytoplasm of macrophages in different groups. ^***^p < 0.001, ^****^p < 0.0001, and ns, no significance by one-way ANOVA. All results are shown as the means ± SEM. Scale bar: 40 μm

Previous findings suggest trehalose inhibits foamy macrophages formation, reduces inflammatory, promotes axonal growth, and aids motor function recovery post-injury. However, its role in enhancing macrophage degradation of engulfed substances is unclear. In recent years, trehalose has been demonstrated to induce autophagy in Alzheimer’s and Huntington’s disease [23, 49]. Autophagy, as a critical pathway for intracellular substance degradation and metabolism, aids in the transfer of endogenous or exogenous cellular materials for degradation within lysosomes [50]. We thus focused on the autophagy and lysosomal function of macrophages after trehalose treatment following SCI, in the control group, immunofluorescence results indicated that, P62^+^ autophagic substances [20, 51] gradually accumulated in the first two weeks post-SCI, while LC3B^+^ autophagosomes [20, 51] and Lamp1^+^ lysosomes-associated membrane proteins decreased, suggesting a progressive decline in autophagic lysosomal function of macrophages in the lesion area over time (Fig. 5A-C, E-G). Trehalose treatment increased autophagy-related protein LC3B and lysosome-related protein Lamp1 levels and reduced P62 expression, indicating that trehalose enhances autophagic lysosomal function in macrophages post-SCI, thereby facilitating intracellular substance degradation (Fig. 5A-C, E-G). TFEB plays a pivotal role in activating autophagy by regulating the expression of autophagy and lysosome-related genes [52]. The results revealed a substantial increase in TFEB^+^ cell numbers and enhanced TFEB nuclear localization in macrophages in the trehalose-treated group compared to the control group (Fig. 5D, H, I). Consistently, further in vitro experiments demonstrated enhanced autophagic lysosomal function (Fig. 6B-D, H) and increased TFEB expression (Fig. 6E-G) after trehalose treatment in RAW 264.7 cells. Accordingly, trehalose may enhance TFEB expression, thereby activating autophagic lysosomal function in macrophages.

### Trehalose treatment may promote the removal of myelin debris by enhancing autophagy

We have demonstrated that the presence of trehalose not only promotes macrophage phagocytosis but also enhances the degradation of phagocytosed substances by macrophages, leading to a decreased formation of foamy macrophages. We hypothesize that this process may be attributed to trehalose enhancing cellular autophagy, thereby promoting macrophage phagocytosis of myelin debris and the procession of neutral lipid droplets from foamy macrophages. To verify this hypothesis, we used a lysosomal inhibitor, chloroquine (CQ), which inhibits the fusion of autophagosomes with lysosomes, thereby inhibiting autophagy [53–55]. Macrophages were incubated in culture media containing myelin debris (0.5 mg/ml), trehalose (20 mM), and chloroquine (30 mg/ml) for 24 h in vitro, followed by ORO staining (Fig. 6A). The results showed that macrophages exhibited significantly reduced myelin debris phagocytic capacity after autophagy was inhibited by chloroquine, similar to the results in the PBS group (Fig. 6I, J). Staining and quantitative analysis were performed using myelin debris labeled with Dio dye and CD68^+^ macrophages, yielding similar results (Fig. 6M, N). Thus, trehalose may promote the phagocytosis of myelin debris by macrophages through the enhancement of autophagy. We further investigated whether trehalose improves the ability of foamy macrophages to expel intracellular lipids by enhancing cellular autophagy. Macrophages were incubated in culture media containing myelin debris (0.5 mg/ml) for 24 h, followed by incubation in media containing trehalose (20 mM) with or without chloroquine (30 mg/ml) for another 24 h (Fig. 6A). The ORO staining results showed that chloroquine abolishes the ability of trehalose to enhance the processing of intracellular lipids by foamy macrophages (Fig. 6K, L). Immunofluorescence and subsequent quantitative analysis yielded similar trends (Fig. 6O, P). Thus, we conclude that trehalose may promote macrophage phagocytosis of myelin debris and the expulsion of lipid droplets from foamy macrophages by enhancing cellular autophagy.

## Discussion

This study reveals that enhancing autophagy post-SCI promotes the removal of myelin debris by macrophages. Trehalose treatment increases TFEB expression and activates autophagy, which not only reduces foamy macrophage formation but also facilitates the processing of neutral lipids within macrophages. Consequently, this accelerates lipid clearance from the lesion area, effectively suppresses inflammation, disrupts scar boundaries, reduces lesion size, and promotes neuron survival, axonal preservation, and motor function recovery (Graphical Abstract). Therefore, trehalose holds promise as a novel therapeutic approach for SCI.

Spinal cord injury is a severe CNS trauma causing motor and sensory dysfunction, leading to significant burdens on patients and families [1, 2]. Facing the global challenge of SCI, there are currently two categories of breakthrough solutions: one stemming from tissue engineering and materials science, and the other from biological approaches. The first includes novel biocompatible electrode materials, brain-machine interfaces, and epidural electrical stimulation technologies that bypass scar barriers. For instance, representative research from Lorach et al.. last year developed a brain-spinal cord interface capable of restoring communication between the brain and spinal cord. However, the stimulation parameters and safety still require further experimental validation [56]. On the other hand, the second category involves the local application of growth factors to inhibit scar formation. Representative achievement comes from Anderson et al.., who utilized a cocktail therapy. Specifically, they overexpressed osteopontin, insulin-like growth factor 1, and ciliary-derived neurotrophic factor in thoracic neurons via AAV virus. And then established a conducive regenerative microenvironment in the injury area using fibroblast growth factor 2 and epidermal growth factor. This approach enabled regenerating axons to successfully cross the injury site and transmit signals, although motor function was not improved [57]. Overall, clinically effective therapeutic approaches remain very limited [58, 59]. Therefore, there is an urgent need to find new, effective therapeutic methods that can be rapidly translated into clinical practice. Trehalose, a safe, non-toxic natural sugar [60] with neuroprotective activity [61], is already widely used in food processing, cosmetics, biotechnology, and pharmaceuticals [62]. Increasing evidence indicates trehalose’s beneficial role in treating neurodegenerative diseases with abnormal protein aggregation, such as Alzheimer’s and Huntington’s diseases [23, 24]. This study found that after SCI, trehalose can effectively reduce inflammatory responses, promote neuron survival, axonal preservation, and motor function recovery. Additionally, trehalose has the advantages of being relatively inexpensive and easy to administer, highlighting its greater potential for clinical application.

After SCI, a substantial number of neural cells undergo necrosis and apoptosis, leading to demyelination in the spinal cord. This results in the accumulation of significant myelin debris, which persists in the injury microenvironment for an extended period [63]. Previous studies have shown that the abnormal accumulation of myelin debris can directly inhibit the formation of growth cones at axon terminals, thereby hindering axonal regeneration [5]. In addition, it can suppress oligodendrocyte maturation, obstruct axonal remyelination [64], mediate the release of pro-inflammatory factors from endothelial cells, exacerbate macrophage infiltration [32], and disrupt tight junctions between endothelial cells, leading to persistent leakage of the blood-spinal cord barrier [65]. The consequences of these factors are catastrophic, severely impeding nerve regeneration and functional recovery. To restore the microenvironment to homeostasis, specialized phagocytes, specifically microglia and macrophages, recognize, engulf, and degrade harmful myelin debris. Compared to macrophages, microglia can secrete chemokines to recruit blood-derived macrophages, coordinating efforts to maintain the stability of the injury microenvironment [2]. Moreover, microglia exhibit superior lipid-processing capabilities, unlike macrophages which become foamy macrophages that occur when macrophages fail to process ingested lipids [41]. Additionally, microglia respond more rapidly to the injury site, arriving within 24 h to phagocytose damaged and degenerating tissues and efficiently process the engulfed material [66]. Research indicates that in the early stages after SCI, microglia primarily perform phagocytic functions, serving as the main phagocytes within the first 3 dpi. Subsequently, peripheral circulating macrophages begin to infiltrate the injured spinal cord. By 7 dpi, these peripheral macrophages become the primary cells responsible for processing degenerated axons and engulfing a larger quantity of material. The persistent presence of phagocytosed substances within the macrophages, which cannot be degraded, causes these cells to gradually transform into foamy macrophages, consequently affecting their survival capacity [40, 67], promoting lipid efflux within foamy macrophages is crucial for spinal cord injury prognosis [68]. Therefore, we focused on macrophages to discover ways to myelin debris clearance to aid spinal cord injury recovery. Our results indicate that the trehalose treatment group had significantly reduced myelin debris in the lesion area at 7 dpi, with all subsequent time points significantly lower than the control group. In the control group, the number of foamy macrophages increased 14 dpi, whereas no significant increase was observed in the trehalose-treated group. This suggests that trehalose treatment reduces foamy macrophage formation and myelin debris accumulation within macrophages. Although we further confirmed the role of trehalose in macrophage phagocytosis using in vitro cell lines, we cannot exclude the possibility that the effects observed in in vivo experiments are indirectly mediated by microglia. This is because recent studies have shown that microglia can influence the formation of foamy macrophages following SCI [69]. Nonetheless, our study preliminarily confirms that trehalose is generally beneficial for the clearance of myelin debris in the microenvironment of SCI. Besides, we mainly studied the increased function of trehalose in autophagy after SCI, but the direct mechanism needs to be further explored. In addition, hyperactive autophagy is thought to lead to apoptosis [70, 71], and it remains to be tested whether enhanced macrophage autophagy can also lead to apoptosis in dysfunctional foamy macrophages, thereby mitigating the inflammatory responses. Additionally, to avoid the increased risk of bladder rupture and urinary tract infections due to urination difficulties, this study exclusively used female mice. Previous SCI studies, including those on macrophage and microglia, commonly employ female animal models [69, 72, 73], the existence of macrophage/microglial sexual dimorphic [74, 75] should not be disregarded. Future research should consider the role of sex in both pathology and treatment.

Autophagy, a crucial intracellular pathway for degrading and metabolizing materials via lysosomal, plays a significant role in various diseases [34, 76]. Although enhanced autophagy has been reported to accelerate neuronal cell death [77, 78], some autophagy inducers, such as betulinic acid, have been shown to enhance macrophage autophagy post-SCI and induce phagocytosis through the AMPK-mTOR-TFEB signaling pathway, thereby eliminating ROS accumulation and promoting injury recovery [27]. Although the role of autophagy in SCI remains controversial, it is a critical cellular process for clearing cytoplasmic waste and damaged organelles [79, 80]. Post-injury, autophagy may contribute to a protective environment, promoting neuronal survival and axonal regeneration [81]. Therefore, autophagy is generally considered to have a beneficial effect in SCI. In atherosclerosis models, trehalose can rescue autophagy-lysosomal dysfunction in foamy macrophages through TFEB overexpression, enhancing lipid phagocytosis and metabolism, reducing inflammation, and delaying plaque formation [20]. Consequently, we hypothesized trehalose improves SCI outcomes by enhancing autophagy. Research suggests that macrophages, as primary phagocytic cells in SCI, are influenced by autophagy [82]. Toll-like receptors (TLRs) are key receptors for macrophage recognition of phagocytic substrates. During TLR pathway activation, autophagy-related proteins like Beclin 1, LC3, and ATG5 are recruited to macrophage phagosomes, a process known as LC3-associated phagocytosis (LAP) [82]. Activating LAP aids innate immunity by recognizing and clearing pathogens via phagocytosis and autophagosome acidification, involving various related proteins [83–85]. Enhancing autophagy may boost macrophage phagocytosis. Phagocytic substrates enter macrophages and are encapsulated by autophagosomes, transported to lysosomes, where they are digested and degraded. Our study’s immunofluorescence staining results suggest improved prognosis linked to enhanced autophagy in macrophages after SCI. We observed P62 accumulation and reduced LC3-labeled autophagosomes and lysosomes at 7 dpi, indicating macrophage autophagic dysfunction. At 14 dpi, significant foamy macrophage formation and myelin debris accumulation occurred in the lesion area. We also examined the upstream autophagy regulator, TFEB [25], whose expression and nuclear translocation increased at 7 dpi following trehalose treatment, potentially explaining the rise in autophagosome and lysosome numbers and the reduction in the P62 autophagy substrate in the trehalose treatment group. In vitro experiments also demonstrate that trehalose enhances macrophage autophagy, and its pro-recovery effects were counteracted by autophagy inhibitors, such as chloroquine. Given the extremely complexity of in vivo environment, we did not perform in vivo rescue experiments to provide more direct evidence for the restoration of autophagy-promoting functions regulated by trehalose, which is a limitation of our study. This study is the first to investigate trehalose’s effects on macrophages in a spinal cord injury model. However, the specific mechanisms by which trehalose enhances TFEB overexpression post-spinal cord injury remain to be further explored.

## Conclusion

In summary, our study demonstrates that trehalose treatment can facilitate the clearance of myelin debris, reduce the formation of foamy macrophages in the lesion area, inhibit the inflammatory responses, decrease the size of fibrotic scars, and promote axonal preservation, neuronal survival, and the recovery of motor functions after SCI. This provides a new perspective for the treatment of SCI.

## Data Availability

The data used and/or analyzed during the current study are available from the corresponding author upon reasonable request.

## Abbreviations

SCI: Spinal cord injury
CNS: central nervous system
TFEB: Transcription factor EB
PFA: paraformaldehyde
DAPI: 4ʹ6-diamidino-2-phenylindole
DSA: donkey serum albumin
ORO: Oil Red O
BMS: The Basso Mouse Scale
Dpi: days post-injury
SEM: standard error of the mean
ECM: extracellular matrix
ANOVA: analysis of variance
